# Repellent and Contact Toxicity of *Alpinia officinarum* Rhizome Extract against *Lasioderma serricorne* Adults

**DOI:** 10.1371/journal.pone.0135631

**Published:** 2015-08-20

**Authors:** Jianhua Lü, Dan Ma

**Affiliations:** School of Food Science and Technology, Henan University of Technology, Lianhua Street, Zhengzhou High-Tech Development Zone, Zhengzhou, 450001, Henan, China; University of Tours, FRANCE

## Abstract

The repellent and contact toxicities of *Alpinia officinarum* rhizome extract on *Lasioderma serricorne* adults, and its ability to protect stored wheat flour from *L*. *serricorne* adults infestation were investigated. The *A*. *officinarum* extract exhibited strong repellent and contact toxicities against *L*. *serricorne* adults. The toxicities enhanced significantly with the increasing treatment time and treatment dose. The mean percentage repellency value reached 91.3% at class V at the dose of 0.20 μL/cm^2^ after 48 h of exposure. The corrected mortality reached over 80.0% at the dose of 0.16 μL/cm^2^ after 48 h of exposure. The *A*. *officinarum* extract could significantly reduce *L*. *serricorne* infestation level against stored wheat flour. Particularly, the insect infestation was nil in wheat flour packaged with kraft paper bags coated with the *A*. *officinarum* extract at the dose of above 0.05 μL/cm^2^. The naturally occurring *A*. *officinarum* extract could be useful for integrated management of *L*. *serricorne*.

## Introduction

The cigarette beetle, *Lasioderma serricorne* (Fabricius) (Coleoptera: Anobiidae), also called as the tobacco beetle, is one of the most destructive insects of many stored food products including flours, dried fruits such as raisins and dates, cocoa, cereals, herbs, spices, nuts, dry pet foods, tobacco and other products worldwide [1–3]. Methyl bromide and phosphine fumigation had been an effective method to control stored product insects in the world [4,5]. However, methyl bromide has been restricted due to its depleting the ozone layer [6,7]. Phosphine fumigation has almost been the only method to control stored product insects [8]. Repeated use of phosphine fumigation for decades has resulted in serious negative effects, such as environmental threat, pesticide residue in food, lethal effects on non-target organisms and insecticide resistance [9–12], which could threaten the future use of phosphine [13,14]. Therefore, it is urgent to develop alternative control methods [4,15]. Plant-derived insecticides might be potential replacement candidates [16–18].

In fact, much effort has been focused on plant-derived materials as alternatives to synthetic pesticides or as lead compounds for many years [19,20]. Moreover, many plant-derived materials have been recently researched as insect-resistant packaging materials based on their strong repellent activity. Natural botanical antifeedants, citronella, protein-enriched pea flour, *Citrus reticulate* oil, *Pimpinella anisum* oil, *Anethum graveolens* oil, *Allium sativum* oil and *Ocimum basilicum* oil, etc. have been confirmed to have strong potential for preventing packaging materials from insects infestation, and some of them are being applied on packaging materials for their effect on avoiding insect penetration [21–24].

*Alpinia officinarum*, a traditional Chinese herbal plant, is widely cultivated in southern China, and its pungent and aromatic rhizome is usually used to treat epigastric pains, nausea, indigestion, duodenal ulcer, gastroenteritis and tinea versicolor infection due to its antioxidant, anti-inflammatory, anticancer, anti-proliferative and antiemetic activities [25–27]. Like other traditional Chinese herbal plants, *A*. *officinarum* has been used as a traditional method by farmers to protect stored grains from insect infestation for many centuries in China [28–30]. Here, we firstly evaluated the potential repellent and contact activities of *A*. *officinarum* rhizome extract against *L*. *serricorne* adults, and its ability to prevent *L*. *serricorne* adults from infestating stored wheat flour in the laboratory.

## Materials and Methods

### Insects

Laboratory cultures of the cigarette beetle, *L*. *serricorne*, were maintained on sterilized diet (wheatfeed/yeast, 95:5, w/w) at 27 ± 2°C, 75 ± 5% r.h. and a 12:12 light:dark photoperiod. Healthy *L*. *serricorne* adults (three–five days old) were used for bioassays.

### Preparation of the extract

The *A*. *officinarum* rhizome is a common Chinese medicine plant grown in China. We bought it from a farmer at Xuwen (20.2335, 110.2110), Guangdong, South China, October 2012. No specific permissions are required for getting *A*. *officinarum* rhizome in China. The *A*. *officinarum* rhizome was identified by the Biology Department of Zhengzhou University, then dried at room temperature and finely ground to powder. Each 50 g of the powder was extracted by Soxhlet method with 250 mL anhydrous diethyl ether (analytical purity) until the distilled liquid was colorless. The solvent was evaporated under vacuum in a rotary evaporator, then the extract (in the remainder of this paper referred to as “*Alpinia* extract”) was stored in airtight fuscous glassware at 4°C.

### Repellency bioassay

The repellent effect of the *Alpinia* extract against *L*. *serricorne* adults was evaluated using the area preference method. Test areas consisted of Whatman No.1 filter paper cut in half (Φ12.5 cm). An aliquot of 1.54, 3.07, 6.14 and 12.28 μL of the *Alpinia* extract dissolved in 1 mL acetone (analytical purity) was evenly applied on half-filter paper discs using a micropipette corresponding to the doses of 0.025, 0.05, 0.10 and 0.20 μL/cm^2^ respectively. The other half of the remaining filter paper was treated with 1 mL acetone alone and used as a control. The filter papers were air-dried for about 5 min to evaporate the solvent completely and full discs were subsequently remade by attaching treated halves to untreated halves with clear adhesive tape. Each remade filter paper disc was tightly fixed on the bottom of a 12.5 cm diameter Petri dish daubed with polytetrafluoroethylene on the inside wall to prevent the insects from escaping. A filter paper disc with both halves treated with 1 mL acetone alone was tested as a blank control. Then 30 unsexed *L*. *serricorne* adults (3–5 days old) were released at the center of the filter paper disc and the Petri dishes were subsequently covered and kept in incubators at 27 ± 2°C, 75 ± 5% r.h. and a 12:12 light:dark photoperiod. Each treatment was replicated four times and the number of insects present on the control (N_c_) and treated (N_t_) areas of the discs was recorded after 12, 24, 36, and 48 h, respectively.

Percentage repellency (PR) values were calculated as follows:

$$PR=[(N_{c}-N_{t})/(N_{c}+N_{t})]100\%$$

The mean percentage repellency value was calculated and assigned to repellency classes from 0 to V [31]: class 0 (PR< 0.1%), class I (PR = 0.1–20%), class II (PR = 20.1–40%), class III (PR = 40.1–60%), class IV (PR = 60.1–80%), class V (PR = 80.1–100%). The extreme PR values express two extreme conditions: 0 showing no repellency, and 100 showing the strongest repellency.

### Contact toxicity

An aliquot of 0.6, 1.2, 2.4 and 4.8 μL of *Alpinia* extract dissolved in 0.4 mL acetone (analytical purity) was evenly applied to a Whatman No.1 filter paper (Φ6 cm) corresponding to the doses of 0.02, 0.04, 0.08 and 0.16 μL/cm^2^, respectively. Applying 0.4 mL acetone alone (the dose of 0 μL/cm^2^) to a Whatman No.1 filter paper (Φ6 cm) was taken as a control. Then, the filter paper was dried in air for 5 min prior to being closely fixed on the bottom of a clean Petri dish (Φ6 cm) by solid adhesive. The Petri dish was in advance daubed with polytetrafluoroethylene on the inside wall to avoid the insects escaping. Thirty treated unsexed *L*. *serricorne* adults (3–5 days old) were introduced into the Petri dish. The Petri dish was covered and kept in incubators at 27 ± 2°C and 75 ± 5% r.h. and the number of dead insects was recorded after 12, 24, 48 and 72 h. Insects showing any movement were considered to be alive when prodded with a camel’s hair brush. Four replicates were conducted.

### Infestation test

Kraft paper bags (80 g/m^2^) and nonwoven cloth bags were made by hand, and their specification was 8 cm × 15 cm. Bags were carefully checked for presence of pores prior to infestation test. The kraft paper bags were sealed with gluewater (Chenguang, Shanghai Chenguang Stationery Co., Ltd.), nonwoven cloth bags were sealed by carefully stitching. Each bag was coated with *Alpinia* extract at the doses of 0 (as a control), 0.025, 0.05, 0.10 and 0.20 μL/cm^2^ respectively. A bag with 50 g of whole wheat flour was put in a glass bottle (500 mL), then 20 unsexed *L*. *serricorne* adults were released into the glass bottle. The *L*. *serricorne* adults were outside of the packaged wheat flour. Subsequently, the glass bottles were covered with pieces of cloth, tied with rubber bands and kept at 27 ± 2°C and 75 ± 5% relative humidity. The number of insects (live larvae and adults) in whole wheat flour was recorded after 45 days. Each treatment was replicated four times.

### Statistical analysis

The percentage mortality was corrected by the Abbott formula [32]. The percentage mortality was determined and transformed to arcsine square-root values for repeated measures analysis of variance (ANOVA). The percentage repellency value of *Alpinia* extract against *L*. *serricorne* adults was also analyzed using repeated measures analysis of variance. The number of *L*. *serricorne* population in whole wheat flour packaged with nonwoven cloth bags and kraft paper bags coated with *Alpinia* extract was analyzed using two-way analysis of variance. Treatment means were compared and separated by Scheffe’s test at *p* = 0.05. The LD_50_ values were calculated using probit analysis. These analyses were performed using SPSS Version 16.0 software.

## Results

### Repellent activity

The repellent activity of *Alpinia* extract progressively increased with increasing exposure dose and exposure period (Table 1), while the *L*. *serricorne* adults randomly moved during the whole testing period in the blank control arenas. The mean percentage repellency value reached 91.3% at class V at the dose of 0.20 μL/cm^2^ within 48 h of exposure (Table 2). The interaction dose × exposure time was not significant at *p*<0.05 level (Table 3).

**Table 1:** Repeated measures analysis of variance between subjects effects for the repellent activity of *Alpinia* extract against *L*. *serricorne* adults at the doses of 0.025, 0.05, 0.10 and 0.20 μL/cm^2^ after 12, 24, 36 and 48 h exposure, respectively.

Source	df	Type III SS	Mean square	F-value	p-value
Dose	3	18351.418	6117.139	17.76	0.0001
Error	12	4132.728	344.394		

**Table 2:** The repellent activity of *Alpinia* extract against *L*. *serricorne* adults. Each datum in the table is percentage repellency (mean ± SE, %). The data in a column followed by different letters indicate significant difference tested by Scheffe’s test at *p* = 0.05. The same as below.

Dose (μL/cm^2^)	Exposure time (h)			
	12	24	36	48
0.025	10.5 ± 3.2a	29.3 ± 5.1a	28.7 ± 7.3a	36.3 ± 4.5a
0.05	18.9 ± 2.6a	31.9 ± 6.1a	56.3 ± 6.2b	67.6 ± 6.4b
0.10	27.7 ± 3.1a	52.2 ± 3.6ab	55.2 ± 4.8b	76.6 ± 8.8bc
0.20	44.2 ± 4.2b	74.6 ± 8.9b	81.4 ± 9.9c	91.3 ± 10.4c

**Table 3:** Repeated measures analysis of variance within subject effects for the repellent activity of *Alpinia* extract against *L*. *serricorne* adults at the doses of 0.025, 0.05, 0.10 and 0.20 μL/cm^2^ after 12, 24, 36 and 48 h exposure, respectively.

Source	df	Type III SS	Mean square	F-value	p-value
Exposure time	3	15605.744	5201.914	53.08	0.0000
Dose × Exposure time	9	1653.606	183.734	1.87	0.0880
Error	36	3528.180	98.005		

### Contact toxicity

The contact toxicity of *Alpinia* extract significantly increased with increasing exposure dose (Table 4). The corrected mortality reached over 80.0% at the dose of 0.16 μL/cm^2^ after 48 h of exposure (Table 5). The LD_50_ value of *Alpinia* extract was 0.05 μL/cm^2^ with the Confidence Interval 95% from 0.02 to 0.08 μL/cm^2^ after 48 h of exposure. The regression line equation of *Alpinia* extract was Y = 7.34 X + 1.80, and the correlation coefficient (r value) was 0.98. The interaction dose × exposure time was not significant at *p*<0.05 level (Table 6).

**Table 4:** Repeated measures analysis of variance between subjects effects for the contact toxicity of *Alpinia* extract against *L*. *serricorne* adults at the doses of 0.02, 0.04, 0.08 and 0.16 μL/cm^2^ after 12, 24, 48 and 72 h exposure, respectively.

Source	df	Type III SS	Mean square	F-value	p-value
Dose	3	49843.386	12460.845	147.15	0.0000
Error	15	1270.200	84.680		

**Table 5:** The contact toxicity of *Alpinia* extract against *L*. *serricorne* adults. Each datum in the table is corrected mortality (mean ± SE, %). Mean ± SE mortality on control Petri dish for different doses ranged from 0.0 ± 0.0 to 4.3 ± 2.3%.

Dose (μL/cm^2^)	Exposure time (h)			
	12	24	48	72
0.02	16.1 ± 3.8a	20.7 ± 4.4a	21.7 ± 4.4a	27.9 ± 2.1a
0.04	30.5 ± 5.2b	41.4 ± 3.9b	41.7 ± 3.9b	43.0 ± 3.6b
0.08	33.9 ± 3.3b	62.9 ± 5.7c	64.4 ± 6.3c	65.8 ± 7.8c
0.16	53.4 ± 6.1c	79.3 ± 8.6d	80.8 ± 9.8d	83.3 ± 9.3d

**Table 6:** Repeated measures analysis of variance within subject effects for the contact toxicity of *Alpinia* extract against *L*. *serricorne* adults at the doses of 0, 0.02, 0.04, 0.08 and 0.16 μL/cm^2^ after 12, 24, 48 and 72 h exposure, respectively.

Source	df	Type III SS	Mean square	F-value	p-value
Exposure time	4	3932.386	1310.795	77.554	0.0000
Dose × Exposure time	12	1930.451	160.871	0.277	0.9913
Error	45	429.082	9.535		

### Infesting test

*Alpinia* extract significantly prevented *L*. *serricorne* adults from infesting packaged wheat flour (Table 7). The higher the treated dose of *Alpinia* extract, the fewer *L*. *serricorne* that occurred in the packaged wheat flour after 45 days storage. Specially, the insect infestation was nil in wheat flour packaged with kraft paper bags coated with *Alpinia* extract at the doses of above 0.05 μL/cm^2^. The interaction dose × packaging bag was significant at *p*<0.05 level (Table 8).

**Table 7:** The number of *L*. *serricorne* population in whole wheat flour packaged with nonwoven cloth bags and kraft paper bags after 45 days storage at 27 ± 2°C and 75 ± 5% relative humidity.

Dose (μL/cm^2^)	Nonwoven cloth bags	Kraft paper bags
0	120.0 ± 18.5a	2.3 ± 0.3a
0.025	31.5 ± 3.3b	0.3 ± 0.3b
0.05	10.6 ± 2.8c	0.0 ± 0.0c
0.10	5.7 ± 3.7d	0.0 ± 0.0c
0.20	3.6 ± 1.3e	0.0 ± 0.0c

**Table 8:** Two-way ANOVA analysis for the number of *L*. *serricorne* in whole wheat flour packaged with nonwoven cloth bags and kraft paper bags after 45 days storage at 27 ± 2°C and 75 ± 5% relative humidity.

Fixed effects	df	F-value	p-value
Dose	4	1165.633	0.0000
Packaging bag	1	11348.64	0.0000
Dose × Packaging bag	4	308.238	0.0000
Error	30		

## Discussion

In the present study, *Alpinia* extract showed strong repellent and contact activities against *L*. *serricorne* adults, and could significantly protect packaged wheat flour from *L*. *serricorne* infestation. Specially, eucalyptol has been determined as the main chemical composition of *A*. *officinarum* essential oil recently [27], so it will be very useful to further evaluate the repellent and toxic effect of eucalyptol and other compositions on *L*. *serricorne*. There are other plant extracts or essential oils with obvious toxicities against *L*. *serricorne* adults. The extracts of *Agastache rugosa* whole plant, *Cinnamomum cassia* bark, *Illicium verum* fruit and *Foeniculum vulgare* fruit as well as horseradish (*Cocholeria aroracia*), mustard (*Brassica juncea*) and cinnamon (*C*. *cassia*) oils have strong fumigant toxicities against *L*. *serricorne* adults [4]. Moreover, many plant extracts and their constituents have been studied to possess potential as alternative compounds to currently used synthetic insecticides for the management of populations of stored-product insects [20,30,33–36].

In addition, the present results showed that *Alpinia* extract was repellent enough to reduce insect immigration into packaged wheat flour when coated on nonwoven cloth bags and kraft paper bags. Furthermore, the *Alpinia* extract is considered to be safe for human being and the environment because it has been a Chinese traditional pharmaceutical agent for generations. Therefore, *Alpinia* extract had the strong potential to be used in the preparation of insect-resistant and biodegradable packaging materials.

Sound packaging material is an important defence line to protect the stored product from insect infestation during the storage period. Most stored-product insects can effectively find wheat flour by the clue of odour emitted from stored wheat flour, and then enter stored-flour by penetrating through the packaging materials or existing holes in the packaging materials [37–40]. Hence, applying insect repellents to food packaging materials has an important practical interest [23].

Usually, insect repellents are often used to improve the packaging material and design for preventing insects from entering packages by modifying the behavior of insects [22,38,41,42]. In fact, some insect repellents have been approved for use as a treatment for insect-resistant packaging in the USA, such as pyrethrins synergized with piperonyl butoxide [43] and methyl salicylate [44]. (E)-2-hexenal, which has potent repellent activity against stored grain insects, is generally used as a flavoring compound by food industries and is commonly recognized as safe by the U.S. Food and Drug Administration [45].

Although the insect infestation of packaged wheat flour treated with *Alpinia* extract has been reduced to the extremely low level in the present study, any infestation of packaged food is unacceptable to consumers. Therefore, it is necessary to determine whether infestation can be completely prevented by using *Alpinia* extract. Of course, the toxic effect of *Alpinia* extract on *L*. *serricorne* and its application on the insect-resistant packaging materials depend on several factors among which are the treatment doses of the plant extract, applied methods and the developing stages of the insect, and so on. Thus, the proper formulation, suitable dose, reasonable application strategy and the effect of environmental factors, as well as composition analysis of *Alpinia* extract deserve to be further researched, so that *Alpinia* extract can be exploited for effectively protecting the stored product from infestations by *L*. *Serricorne* in practice.
